# Alternative Strategy for a Quadrivalent Live Attenuated Influenza Virus Vaccine

**DOI:** 10.1128/JVI.01025-18

**Published:** 2018-10-12

**Authors:** Zhimin Wan, Stivalis Cardenas Garcia, Jing Liu, Jefferson Santos, Silvia Carnaccini, Ginger Geiger, Lucas Ferreri, Daniela Rajao, Daniel R. Perez

**Affiliations:** a Department of Population Health, Poultry Diagnostic and Research Center, College of Veterinary Medicine, University of Georgia, Athens, Georgia, USA; Icahn School of Medicine at Mount Sinai

**Keywords:** LAIV, influenza virus quadrivalent vaccine, protection efficiency, influenza virus vaccines, pandemic influenza, seasonal influenza, universal vaccine, universal vaccine platform

## Abstract

Influenza virus infections continue to pose a major public health threat worldwide associated with seasonal epidemics and sporadic pandemics. Vaccination is considered the first line of defense against influenza. Live attenuated influenza virus vaccines (LAIVs) may provide superior responses compared to inactivated vaccines because the former can better elicit a combination of humoral and cellular responses by mimicking a natural infection. Unfortunately, during the 2013–2014, 2014–2015, and 2015–2016 seasons, concerns emerged about the effectiveness of the only LAIV approved in the United States that prevented the Advisory Committee on Immunization Practices (ACIP) from recommending its use. Such drawbacks open up the opportunity for alternative LAIV strategies that could overcome such concerns. Previously, we developed a combined strategy of temperature-sensitive mutations in the PB2 and PB1 segments and an epitope tag in the C terminus of PB1 that effectively attenuates influenza A viruses of avian and mammalian origin. More recently, we adopted a similar strategy for influenza B viruses. The resulting attenuated (*att*) influenza A and B viruses were safe, immunogenic, and protective against lethal influenza virus challenge in a variety of animal models. In this report, we provide evidence of the potential use of our *att* strategy in a quadrivalent LAIV (QIV) formulation carrying H3N2 and H1N1 influenza A virus subtype viruses and two antigenic lineages of influenza B viruses. In naive DBA/2J mice, two doses of the QIV elicited hemagglutination inhibition (HI) responses with HI titers of ≥40 and effectively protected against lethal challenge with prototypical pandemic H1N1 influenza A and influenza B virus strains.

**IMPORTANCE** Seasonal influenza viruses infect 1 billion people worldwide and are associated with ∼500,000 deaths annually. In addition, the never-ending emergence of zoonotic influenza viruses associated with lethal human infections and of pandemic concern calls for the development of better vaccines and/or vaccination strategies against influenza virus. Regardless of the strategy, novel influenza virus vaccines must aim at providing protection against both seasonal influenza A and B viruses. In this study, we tested an alternative quadrivalent live attenuated influenza virus vaccine (QIV) formulation whose individual components have been previously shown to provide protection. We demonstrate in proof-of principle studies in mice that the QIV provides effective protection against lethal challenge with either influenza A or B virus.

## Abstract (precis)

Seasonal influenza viruses infect 1 billion people worldwide and are associated with ∼500,000 deaths annually. In addition, the never-ending emergence of zoonotic influenza viruses associated with lethal human infections and of pandemic concern calls for the development of better vaccines and/or vaccination strategies against influenza virus. Regardless of the strategy, novel influenza virus vaccines must aim at providing protection against both seasonal influenza A and B viruses. In this study, we tested an alternative quadrivalent live attenuated influenza virus vaccine (QIV) formulation whose individual components have been previously shown to provide protection. We demonstrate in proof-of principle studies in mice that the QIV provides effective protection against lethal challenge with either influenza A or B virus.

## INTRODUCTION

Type A (IAV) and type B (IBV) influenza viruses, within the family Orthomyxoviridae (1), are responsible for yearly epidemics of respiratory disease in humans. The Word Health Organization (WHO) estimates that seasonal influenza virus infections result in about 1 billion infections, 3 to 5 million cases of severe disease, and between 300,000 and 500,000 deaths around the world every year (2). For the United States, influenza virus infections result in an average economic impact of $87 billion due to prophylactic, therapeutic, and hospitalization costs, as well as missed school days or workdays (3–5). IBVs contribute less to seasonal epidemics than IAVs, but they are a significant disease burden in the pediatric and elderly populations (6–8). In addition, IAVs have been associated with pandemic episodes when novel strains from animal reservoirs are introduced into humans (9). Public health concerns are aggravated by the inherent ability of influenza viruses to develop resistance to available antivirals (10). Thus, vaccination remains the best choice to protect humans against influenza virus infections, and it is considered the first line of defense against influenza virus-associated illness.

Vaccines against seasonal influenza viruses are manufactured to confer protection against circulating IAV and IBV strains. Current vaccines rely primarily on antibody responses to the virus' hemagglutinin (HA) surface protein. However, HA undergoes antigenic drift, requiring regular vaccine updates in order to antigenically match them to the currently circulating strains (11, 12). Seasonal influenza virus vaccines have traditionally contained three influenza virus strains, two IAV strains (A/H1N1 and A/H3N2) and one IBV from either the B/Yamagata or the B/Victoria antigenic lineage (11). In recent years, however, the two IBV lineages have shown not only seasonal variations, but also significant differences in prevalence in different countries. Thus, the U.S. Food and Drug Administration (FDA) has approved quadrivalent vaccines that incorporate both IBV antigenic lineages, in addition to the two IAV strains (13, 14). To date, the FDA has approved three types of influenza virus vaccines for human use: inactivated influenza virus vaccine (IIV), recombinant influenza protein (RIP) vaccine, and live attenuated influenza virus vaccine (LAIV) (15, 16). The most widely used influenza virus vaccine is IIV, which can elicit protective humoral immunity by inducing the production of neutralizing antibodies that target epitopes on the virus HA (and, to a lesser extent, the neuraminidase [NA] surface protein). The RIP vaccine, similar to IIV, can induce neutralizing antibodies that target the HA protein (17). In contrast, LAIVs can elicit not only humoral but also cell-mediated immunity, which is considered more cross-protective, since it targets more conserved viral epitopes (18, 19). LAIVs for human use were independently obtained by serial passage in eggs at low temperatures, resulting in cold-adapted (*ca*) and temperature-sensitive (*ts*) mutations that resulted in an attenuated (*att*) phenotype in *vivo*. In the United States, the only FDA-approved LAIV consists of viruses carrying a series of mutations in the internal gene segments of the master donor viruses (MDVs) (A/Ann Arbor/6/60 [H2N2] [MDV-A] and B/Ann Arbor/1/66 [MDV-B]) (20–22).

Early work in our laboratory demonstrated that a combination of an HA tag at the C terminus of PB1 and *ts* mutations in PB2 (S265) and PB1 (E391, G581, and T661) showed remarkable stability over multiple passages in IAV backbones of avian, human, and swine origin. This strategy—referred to as IAV *att*—is safe and effective, resulting in protection against avian- and mammalian-origin influenza virus strains in a variety of avian and mammalian species (23, 24). We recently demonstrated that an IBV *att* strain modified with a similar strategy (PB1 with mutations G580, A660, and a C-terminal HA tag) resulted in a stable virus over multiple passages in tissue culture and eggs and with an *att* phenotype *in vivo*. A single intranasal (i.n.) dose of the IBV *att* protected mice against lethal challenge with antigenically matched and mismatched IBV strains (25). In this study, we used the IAV *att* and IBV *att* backbones to generate a quadrivalent LAIV (QIV) containing H3N2 and H1N1 IAV antigenic subtypes and two antigenic IBV lineages. We used the QIV to immunize mice intranasally in a prime-boost regime. We show that 2 doses of the QIV elicited discernible hemagglutination inhibition (HI) responses against the 4 viruses in the vaccine. We also observed that the QIV protected mice against lethal challenge with either IAV H1N1 or IBV.

## RESULTS

### Generation of a QIV.

In this study, we sought to evaluate whether a QIV with the two laboratory *att* backbones was safe, immunogenic, and protective. Thus, reverse genetics was used to generate the following viruses: Ty/04 *att* (H3N2), Ca/04 *att* (H1N1), B/Bris *att* (Victoria lineage), and B/Wisc *att* (Yamagata lineage). The Ty/04 *att*, Ca/04 *att*, and B/Bris *att* viruses have been previously described (23–25), while the B/Wis *att* virus was prepared for this study (Table 1). Like the B/Bris *att* virus, the B/Wis *att* virus displayed a *ts* phenotype *in vitro* that is typically associated with attenuation *in vivo* (data not shown). The QIV formulation was prepared with doses containing equal amounts (2 × 10^7^ 50% tissue culture infectious doses [TCID_50_]/ml) of each *att* virus.

**TABLE 1:** Influenza viruses used in this study

Virus name	Description	Designated abbreviation	Reference
A/turkey/Ohio/313053/2004 (H3N2)	Wild-type A/turkey/Ohio/313053/2004 generated by reverse genetics	Ty/04	24
ma-A/California/04/2009 (H1N1)	Mouse-adapted A/California/04/2009 generated by reverse genetics	Ca/04	42
B/Brisbane/60/2008 PB2 F406Y	B/Brisbane/60/2008 carrying the PB2 Y406 mutation generated by reverse genetics	B/Bris	25
B/Wisconsin/01/2010	Wild-type B/Wisconsin/01/2010	B/Wisc	25
A/turkey/Ohio/313053/2004 (H3N2) *att*	*att* virus Ty/04 with PB1 E391, G581, T661, HA tag, and PB2 S265 mutations; other gene segments from wild-type Ty/04; generated by reverse genetics	Ty/04 *att*	24
A/California/04/2009 (H1N1) *att*	Reassortant *att* virus with PB1 and PB2 gene segments from Ty/04 *att*; other gene segments from ma-Ca/04; generated by reverse genetics	Ca/04 *att*	This study
B/Brisbane/60/2008 *att*	B/Brisbane/60/2008 with PB1 G580, A660, and HA tag; other gene segments from unmodified wild-type B/Bris strain (PB2 F406); generated by reverse genetics	B/Bris *att*	25
B/Wisconsin/01/2010 *att*	Reassortant *att* virus carrying HA and NA gene segments from B/Wisc and remaining genes from B/Bris *att*; generated by reverse genetics	B/Wisc *att*	This study

### Safety and immunogenicity of QIV.

**FIG 1:**
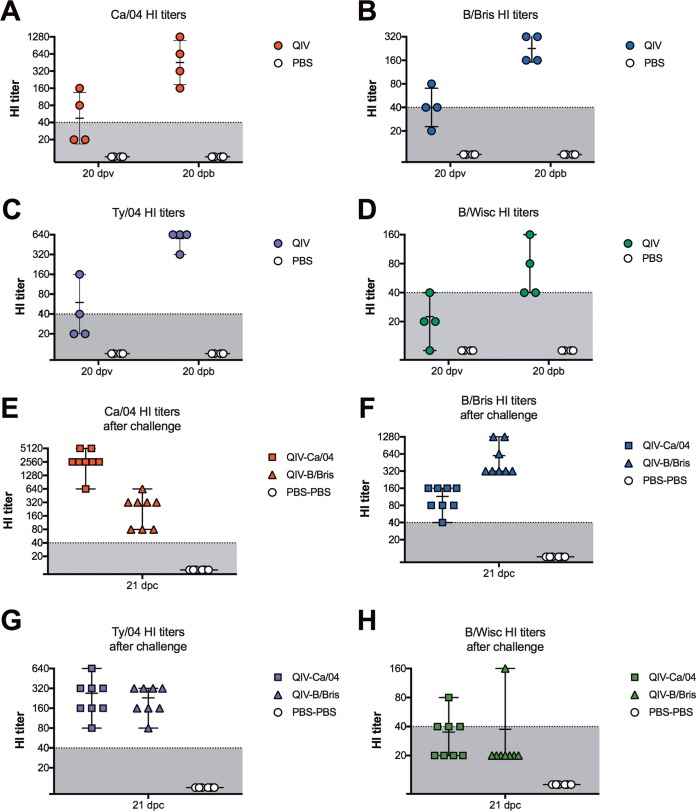
HI titers against vaccine viruses. Shown is titration of antibodies against each virus by HI assays after vaccination and challenge. Six-week-old female DBA/2J mice were inoculated i.n. with QIV containing Ty/04 *att*, Ca/04 *att*, B/Bris *att*, and B/Wis *att* viruses. The mock-vaccinated control group received PBS. At 21 dpv, the QIV-vaccinated mice were boosted with a second dose of the QIV, whereas the PBS control mice were mock vaccinated. At 21 dpb, the mice were challenged with a lethal dose of either the Ca/04 (QIV-Ca/04 and PBS-Ca/04 groups) or B/Bris (QIV-B/Bris and PBS-B/Bris) virus. A third group was mock challenged with PBS (PBS-PBS group). (A to D) Serum HI titers against each of the homologous viruses in the QIV measured at 20 dpv and 20 dpb. (E to H) Serum HI titers against each of the homologous viruses in the QIV at 21 dpc with either Ca/04 or B/Bris. Horizontal bars indicate samples collected at 21 dpc. Error bars correspond to median and range.

DBA/2J mice were randomly distributed into two different groups and received either the QIV or phosphate-buffered saline (PBS) (mock vaccination) by intranasal inoculation. The mice were boosted at 21 days postvaccination (dpv) following the same strategy. No clinical signs of disease, body weight loss, or mortality were observed after prime or boost. HI titers were evaluated at 20 dpv and 20 days postboost (dpb) from 4 mice/group (Fig. 1). Mice in the QIV group showed low but discernible HI titers at 20 dpv against the four HA components in the vaccine (HI titers of 20 to 160 against Ca/04, 20 to 80 against B/Bris, 20 to 160 against Ty04, and 10 to 40 against B/Wisc). After boost, HI titers increased and were all within the range considered to be predictive of protection against clinical disease (HI titers of 160 to 1,280 against Ca/04, 160 to 640 against B/Bris, 320 to 640 against Ty/04, and 40 to 160 against B/Wisc). As expected, mice in the PBS group showed HI titers below the limit of detection. These results indicated that the QIV was safe and immunogenic in mice.

### Protection efficacy for QIV in mice.

**FIG 2:**
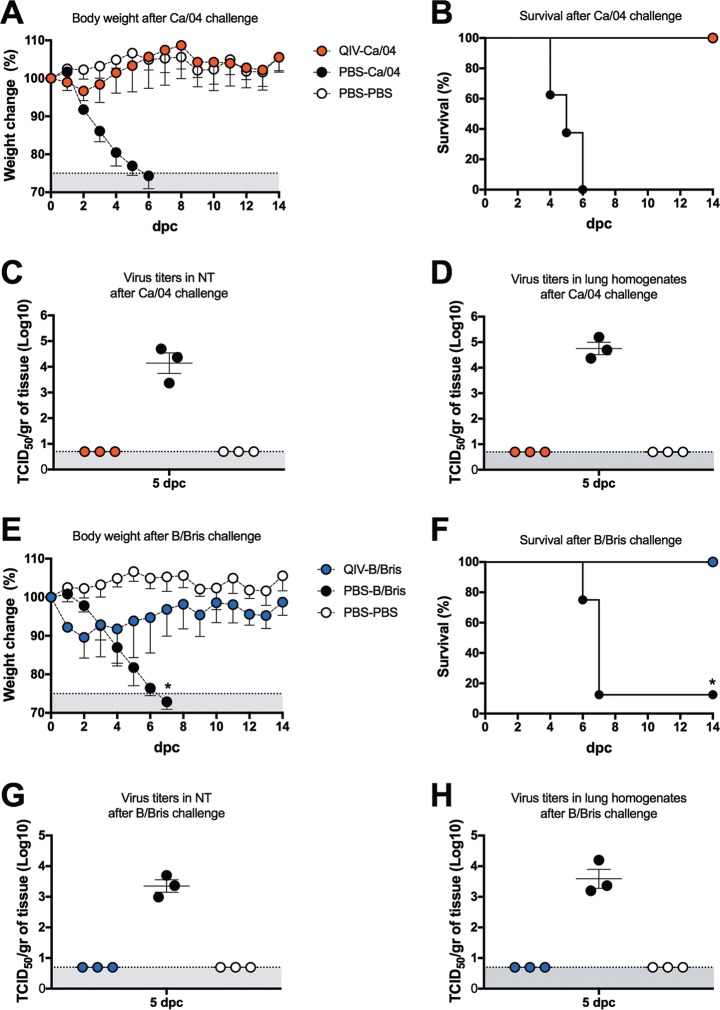
Protective efficacy of QIV against lethal challenge with the Ca/04 or B/Bris virus. (A, B, E, and F) Percentages of change in body weight and survival following challenge with either Ca/04 (A and B) or B/Bris (E and F) virus. (C, D, G, and H) At 5 dpc, a subset of mice (*n* = 3/group) were sacrificed, and NTs and lungs were collected. Virus titers in NTs (C and G) and lung homogenates (D and H) were measured by TCID_50_ in MDCK cells. (E and F) The asterisks denote one mouse outlier in the PBS/B/Bris group that did not show body weight loss, probably due to imperfect inoculation of the B/Bris challenge strain. The same mouse was the only one that survived the challenge with the B/Bris strain. The error bars indicate standard deviation.

In order to assess the protective effects of the QIV regime, mice were challenge with 10^7^ TCID_50_ per mouse of either the Ca/04 or B/Bris virus by the intranasal route (Fig. 2). No signs of disease and minor weight loss were observed in the QIV-vaccinated mice within the first 2 days after challenge with the Ca/04 virus (QIV-Ca/04), but the mice quickly recovered from day 3 onward. All of the QIV-Ca/04 mice (8 out 8) survived the challenge by 14 days postchallenge (dpc). In contrast, the PBS-Ca/04 mice showed rapid body weight loss and weakened after challenge and succumbed or had to be euthanized by 6 dpc. Despite not showing overt clinical signs of disease, the mice in the QIV-B/Bris group showed substantial weight loss (10 to 15%) within the first 3 days after challenge and slowly recovered. By 10 dpc, the mice in the QIV-B/Bris group showed average body weights similar to those of unchallenged control mice. By 14 dpc, all the mice (8 out of 8) in the QIV-B/Bris group had survived the challenge. By comparison, the body weights of mice in the PBS-B/Bris group declined rapidly, and by day 7, 7 out of the 8 mice had succumbed to the challenge. It must be noted that one mouse in the PBS-B/Bris group appeared not to have been infected properly because it showed no body weight loss during the follow-up at 14 days postchallenge (and thus, it was not considered for the calculation of body weight changes in the group). The same mouse is shown as a survivor in the survival curve (an asterisk is used to denote the outlier in Fig. 2E and F). The protective effects of the QIV were also reflected by the lack of detectable virus in lung homogenates and nasal turbinates (NTs) collected at 5 dpc (Fig. 2). Conversely, significant virus titers were observed in lung and NT homogenates obtained from mice in the PBS-Ca/04 and PBS-B/Bris control groups. As expected, HI titers at 21 dpc against the challenge virus were increased in each group but remained unchanged (or slightly lower than at 20 dpb) against other viruses in the vaccine (Fig. 1). These observations suggest that the QIV protected the mice against lethal IAV H1N1 or IBV (Victoria lineage) challenge.

### Virus-specific IgA and IgG antibody responses in mice after challenge.

**FIG 3:**
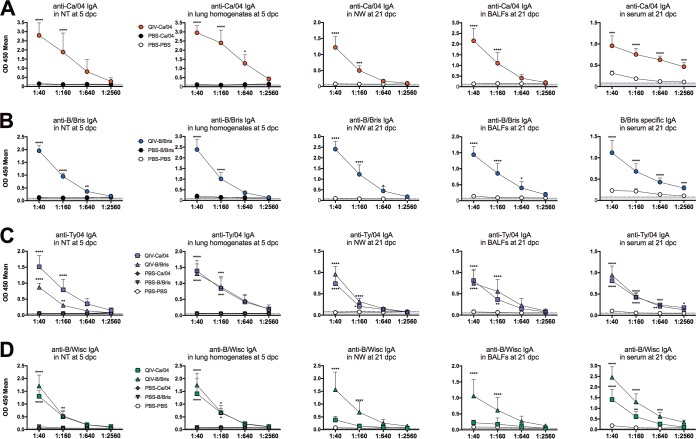
Virus-specific mouse IgA antibodies against homologous viruses in QIV after challenge. Lungs and NTs were collected at 5 dpc; NW, BALFS, and sera were collected at 21 dpc. Shown are virus-specific IgA antibodies against Ca/04 (A), B/Bris (B), Ty/04 (C), and B/Wisc (D) by ELISA. Sample dilutions are shown on the *x* axes. *P* values using two-way ANOVA are indicated for IgA differences between vaccination-challenge groups and the PBS-PBS group. *, *P* < 0.05; **, *P* < 0.01; ***, *P* < 0.001; ****, *P* < 0.0001. The error bars indicate standard deviation. Values on the *x* axis correspond to dilutions of the original samples in PBS prior to testing.

**FIG 4:**
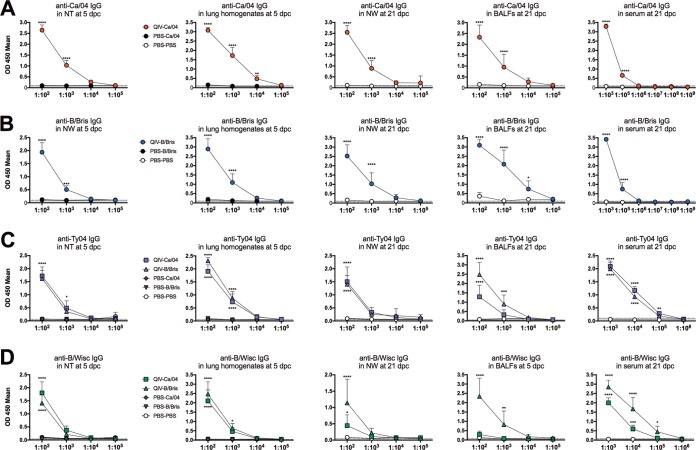
Virus-specific mouse IgG antibodies against homologous viruses in the QIV after challenge. The samples collected are described in the legend to Fig. 3 and in Materials and Methods. Virus-specific IgG antibodies against Ca/04 (A), B/Bris (B), Ty/04 (C), and B/Wisc (D) by ELISA. Sample dilutions are shown on the *x* axes. *P* values using two-way ANOVA are indicated for IgG differences between vaccination-challenge groups and the PBS-PBS group. *, *P* < 0.05; **, *P* < 0.01; ***, *P* < 0.001; ****, *P* < 0.0001. The error bars indicate standard deviations.

To further explore virus-specific antibody responses, we evaluated IgA and IgG levels against the 4 viruses in the vaccine. Enzyme-linked immune absorbent assays (ELISAs) using whole-virus-coated plates were performed to detect IgA and IgG antibodies in samples from lungs and NTs collected at 5 dpc and sera, nasal washes (NW), and bronchoalveolar lavage fluids (BALFs) collected at 21 dpc. Optical density at 450 nm (OD_450_) values ≥2-fold above background were considered positive for virus-specific antibodies. Consistently, virus-specific IgA antibodies against the 4 vaccine viruses were detected in all samples diluted between 1:40 and 1:160 (or even at higher dilutions, depending on the virus and the samples) (Fig. 3). The samples collected at 5 dpc from the QIV-vaccinated mice (QIV-Ca/04 and QIV-B/Bris groups) had absorbance readings well above those of negative-control samples (mock vaccinated and mock challenged [PBS-PBS]) or samples obtained from unvaccinated mice challenged with either Ca/04 or B/Bris virus (PBS-Ca/04 and PBS-B/Bris, respectively), strongly indicating that the QIV had stimulated IgA responses in the mice (Fig. 3). Based on OD_450_ values, IgA responses against the Ca/04 and B/Bris challenge viruses were high in NTs and lung homogenates at 5 dpc and remained high in BALFs and NW at 21 dpc compared to PBS-PBS control group samples (note that mock-vaccinated mice did not survive beyond 7 dpc, except for 1 mouse in the PBS-B/Bris group). As expected, virus-specific IgA levels were also high in fecal samples (not shown) but moderate in serum samples collected at 21 dpc. Similarly, IgG responses were relatively high against the challenge viruses in samples from QIV-vaccinated mice collected after challenge (Fig. 4). Typically, virus-specific IgG was consistently detected well above background in samples diluted between 1:100 and 1:1,000 (up to 1:100,000 in serum) from QIV-vaccinated/challenged mice but not in similarly diluted samples from mock-vaccinated/challenged mice.

Ty/04- and B/Wisc-specific IgA and IgG absorbance readings provided some evidence of the relative levels of QIV-induced responses. However, it must be noted that the samples were collected after challenge with either Ca/04 or B/Bris virus, and the whole-virus ELISAs do not discriminate between the potential postchallenge stimulation of specific anti-Ty/04 or anti-B/Wisc antibodies versus cross-reactive antibodies and/or antibodies against more conserved antigens. Stimulation of antibodies against conserved antigens helps to explain the higher levels of IgA against the Ty/04 virus at 5 dpc in NTs from the QIV-Ca/04 mice than in NTs from the QIV-B/Bris mice. Likewise, IgA and IgG responses against the B/Wisc virus were higher in samples collected at 21 dpc from the QIV-B/Bris mice than in those from the QIV-Ca/04 mice. However, in the remaining samples, the IgA and IgG responses were similar among QIV-vaccinated mice. Thus, the anti-Ty/04 IgA levels in lung homogenates at 5 dpc and in NW, BALFs, and serum at 21 dpc and the anti-Ty04 IgG levels in all the samples (except BALFs at 21 dpc) were similar among mice in the QIV-Ca/04 and QIV-B/Bris groups. Likewise, anti-B/Wisc IgA and IgG responses were similar among QIV-vaccinated mice in 5-dpc NT and lung homogenate samples. Interestingly, BALF samples collected at 21 dpc showed a higher anti-Ty04 IgG response in samples obtained from the QIV-B/Bris group than in those from the QIV-Ca/04 group. These results indicated that mice immunized two times with the QIV produced virus-specific IgA and IgG antibodies. Challenge with the Ca/04 or B/Bris virus resulted in stimulation of a combination of virus-specific antibodies and antibodies against conserved viral antigens.

## DISCUSSION

Influenza virus vaccine effectiveness ranged from 19% to 48% during the past three influenza seasons (26, 27). During the 2013–2014, 2014–2015, and 2015–2016 seasons, a number of issues emerged that called into question the effectiveness of the only FDA-approved LAIV in the U.S. market (no evidence of similar problems appears to have existed in other markets where the same vaccine is approved). Although such issues appear to have been resolved for the 2017–2018 season and alternative LAIVs approved for other markets (based on the A/Leningrad and B/USSR MDV strains) do not seem to have faced similar issues, there is a need for the development of novel LAIV strategies that will likely impact the future development of influenza virus vaccines. In general, weakened (attenuated) forms of viral pathogens offer the longest-lasting protection because they produce multidimensional responses by stimulating the humoral and cellular arms of the immune system. The IAV *att* component in the QIV studied here, based on the internal gene segment constellation of the Ty/04 strain, contains mutations found in the A/Ann Arbor MDV-A (PB2 S265 and PB1 E391, G581, and T661). These mutations can confer an *att* phenotype on some, but not all, IAV strains, as we have previously shown (23, 24, 28, 29). However, the incorporation of the HA epitope tag at the C terminus of PB1, along with the *ts* mutations (*ts* + HA tag = *att*) resulted in attenuation *in vivo*. The IAV *att* strains that we have produced so far are remarkably stable and show optimal safety and protective profiles when used as monovalent vaccines. The most widely used MDV-As (A/Leningrad/and A/Ann Arbor) were isolated nearly 60 years ago. Over this period, IAVs have continued to evolve and reassort and have accumulated mutations on relevant cytotoxic T lymphocyte (CTL) epitopes that differ from those in the MDV-A internal backbones (30). Altering the gene constellation of MDV-A would require further preclinical and clinical testing. Thus, a major advantage of our strategy is the possibility to impart the *att* phenotype to virtually any IAV strain. We favored the Ty/04 *att* backbone because it carries the potential to act as a universal *att* backbone for use in avian and mammalian species, including humans. To be used in humans, however, a complementary IBV *att* strategy is highly desirable. For that reason, we incorporated an IBV PB1 gene segment carrying the G580 and A660 mutations and the C-terminal HA tag and established that it resulted in attenuation of the B/Bris strain. We further demonstrated that the B/Bris *att* strain was stable, safe, and immunogenic and protected mice against lethal IBV challenge. The modifications of the IBV *att* are unique to our virus and are not shared with the B/Ann Arbor or B/USSR MDV-B.

In this study, we evaluated whether a QIV version of the *att* strategy was safe, immunogenic, and protective against lethal influenza virus challenge in a mouse model. We used DBA/2J mice because they are more susceptible to IAV and IBV strains and develop anti-influenza humoral responses comparable to those of other mouse strains (31, 32). We opted for a prime-boost strategy in order to determine whether we could further stimulate humoral responses against all the components of the vaccine. Such a 2-dose strategy (1 month apart) is recommended for children 2 to 8 years old who receive the LAIV in the United States. It must be noted that, except for the Ca/04 *att* strain, the viral components in the QIV do not carry mouse-adapted mutations. Thus, it was remarkable to realize that a second dose of the QIV stimulated HI responses to all components in the vaccine to within the protective range (HI titers of ≥40) (Fig. 1). Differences in the levels of HI responses, particularly the B/Wisc *att*, could be due to a number of factors, including replication fitness differences, competition between IAV and/or IBV strains, and antigenic/immunogenic differences. Further studies, including refinements in virus particle versus infectious dose, will be needed but are beyond the scope of the present report.

QIV-vaccinated mice were fully protected against lethal challenge (∼10,000 50% mouse lethal dose [MLD_50_]), with the Ca/04 strain showing minor body weight losses within the first 2 days after challenge (Fig. 2). The Ca/04 strain used in this study carries mutations on the surface and internal gene segments that increase its virulence for mice (42). However, by 5 dpc, no evidence of Ca/04 replication was detected in the lungs or NTs of the QIV-vaccinated/challenge mice, unlike the mock-vaccinated/challenged mice. QIV-vaccinated mice also survived challenge with the B/Bris strain carrying the PB2 F406Y mutation and by 5 dpc showed no evidence of virus replication in lungs and NTs. We have previously shown that the PB2 F406Y mutation increases the virulence for mice of the wild-type B/Bris strain (it also slightly increases the virulence of B/Wisc) (25). Unexpectedly, the B/Bris challenge dose (≤10 50% lethal doses [LD_50_]) was sufficient to cause significant body weight loss in the QIV-vaccinated/challenged mice compared to the PBS-PBS mice. It is possible that the IAV *att* strains had a competitive advantage over the IBV *att* strains in the QIV that affected humoral and cellular responses against the latter. Whether the effect is due to the mouse model or to true competitive-fitness advantages of one virus over the rest in the QIV remains to be determined. The potential for interference between type A and type B live attenuated influenza viruses has been previously described in the mouse model, although early studies did not contemplate the use of QIV formulations (33, 34). As expected, by 21 dpc, HI titers were increased against the challenge viruses, but only in their respective groups, whereas HI titers against other viruses were slightly lower than at 20 dpb. It was also noted that the HI responses to the H3 HA proteins were limited to the Ty/04 strain with very limited cross-reactivity to other older or newer seasonal H3 strains (not shown).

LAIVs have been shown to heighten innate immune responses (35–37) and to stimulate cross-protective responses to heterologous or antigenically divergent strains (38, 39). We explored anti-IAV and anti-IBV IgA and IgG responses using whole-virus preparations in an ELISA. NTs and lung homogenate samples collected at 5 dpc provided an indication of preexisting immunity against all the components of the QIV. Although direct comparisons are not possible because the 4 viruses in the QIV are different, a trend toward higher OD values for IgA and IgG against the Ca/04 and B/Bris strains than against the Ty/04 and B/Wisc strains was observed, consistent with the first two being the viruses used for challenge. It is expected that cross-protective antibodies and/or antibodies against conserved proteins are represented at some level in the ELISAs, and this appears to be the case for the IBV strains. However, it is important to emphasize that anti-influenza responses are skewed toward the HA and NA proteins. Thus, it is tempting to speculate that the IgA and IgG profiles observed are bona fide representations of antibody responses against each of the viruses in the vaccine (and cross-reactive IBV HA and/or NA antibodies).

In summary, we provide proof of principle of the potential of an alternative QIV as a safe and effective vaccine against IAV and IBV in mice. The strategy increases the arsenal of LAIV options against seasonal and pandemic influenza.

## MATERIALS AND METHODS

### Cells and viruses.

Human embryonic kidney 293T and Madin-Darby canine kidney (MDCK) cells were grown in Dulbecco's modified Eagle's medium (DMEM) (Sigma, St. Louis, MO) supplemented with 10% fetal bovine serum (FBS) (Sigma, St. Louis, MO) and 1% antibiotic/antimycotic (Sigma-Aldrich, St. Louis, MO). The cells were propagated at 37°C in 5% CO_2_. The mouse-adapted pandemic-origin A/California/04/2009 (H1N1) (Ca/04), the swine-like/human-like A/turkey/Ohio/303153/2004 (H3N2) (Ty/04), the B/Brisbane/60/2008 with the PB2 F406Y mutation that increases virulence in mice (B/Bris), and the B/Wisconsin/01/2010 (B/Wisc) strains have been previously described and are referenced in Table 1. The virus stocks were amplified in 9-day-old specific-pathogen-free (SPF) embryonated chicken eggs and were stored at −80°C.

### Generation of *att* viruses by reverse genetics.

The Ty/04 *att* (H3N2) and B/Bris *att* virus vaccines have been previously described and are referenced in Table 1. The Ca/04 *att* (H1N1) virus carries the PB1 and PB2 segments from the Ty/04 *att* virus and 6 gene segments from the mouse-adapted Ca/04 virus. B/Wisc *att* carries the HA and NA surface gene segments of B/Wisc and the backbone of B/Bris *att*. The *att* viruses were rescued by reverse genetics using coculture of 293T and MDCK cells as previously described (40). The IAV *att* and IBV *att* rescued viruses (passage 0 [P0]) were expanded in fresh MDCK cells to produce P1 viruses. The P1 viruses were used in a second round of amplification in MDCK cells (P2) and in 9-day-old SPF eggs (E1). The IAV *att* and IBV *att* viruses were amplified in eggs at 35°C and 33°C, respectively. Virus stocks were titrated by TCID_50_ using the Reed-Muench method (43). The virus stocks were sequenced by next-generation sequencing (NGS) using the MiSeq platform (Illumina, San Diego, CA). The NGS results showed integrity of *att* mutations and absence of spurious mutations. The QIV was formulated to contain equal doses (2 ×10^7^ TCID_50_/ml) of Ty/04 *att*, Ca/04 *att*, B/Bris *att*, and B/Wisc *att* viruses.

### Animal use and compliance.

Animal studies were performed under animal biosafety level 2 (ABSL2) containment conditions and followed protocols approved by the Institutional Animal Care and Use Committees (IACUC) at the University of Georgia. Mice that lost ≥25% of their initial body weight (a score of 3 or higher on a 4-point scale of disease severity) were humanely euthanized.

### Mouse experiments.

Five- to 6-week-old female DBA/2J mice were purchased from Jackson Laboratories (Bar Harbor, ME). The mice were randomly distributed into 2 groups. The mice were anesthetized with isoflurane and subsequently inoculated via the i.n. route with 50 μl of an inoculum containing either PBS (mock vaccinated; group 1; *n* = 33) or QIV (10^6^ TCID_50_/mouse; group 2; *n* = 22). At 21 dpv, the mice in group 2 were boosted i.n. with QIV, whereas the control mice in group 1 received PBS. At 20 dpv and 20 dpb, 4 mice/group were bled from the submandibular vein, and serum samples were collected and used to measure neutralizing and virus-specific antibody responses. At 21 dpb, mice were challenged with an inoculum of 50 μl containing either the Ca/04 virus (10^7^ TCID_50_/mouse; ∼10,000 MLD_50_; *n* = 11/group; groups QIV-Ca/04 and PBS-Ca/04) or the B/Bris virus (10^7^ TCID_50_/mouse; ∼10 MLD_50_; *n* = 11/group; groups QIV-B/Bris and PBS-B/Bris). A third group of mice from the mock-vaccinated group were mock challenged with PBS (*n* = 11; group PBS-PBS). After challenge, the mice were monitored daily for clinical signs of disease, body weight loss, and mortality. At 5 dpc, 3 mice per group were sacrificed, and lungs and NT samples were collected to determine virus titers. At 21 dpc, all the mice were sacrificed, and serum samples, NW, and BALF samples were collected for evaluating virus-specific antibody responses.

### HI assay.

Serum samples collected at 20 dpv, 20 dpb, and 21 dpc were screened for the presence of neutralizing antibodies by the HI assay using the wild-type Ty/04, Ca/04, B/Bris, and B/Wisc strains (41). Briefly, the sera were treated with receptor-destroying enzyme at 37°C overnight and then heat inactivated at 56°C for 30 min. Then, the sera were diluted 1:10 with PBS and subsequently serially diluted 2-fold and mixed with 8 hemagglutination units (HAU) of virus in a 96-well plate and incubated at room temperature for 15 min. The HI activity was visualized by adding 0.5% turkey red blood cells to the virus-serum mixtures and further incubation at room temperature for 30 min before reading.

### Detection of virus-specific IgA and IgG antibodies.

NW samples, BALFs, and serum samples collected at 21 dpc, as well as NT and lung homogenates prepared at 5 dpc, were screened for the presence of virus-specific IgA and IgG antibodies using an ELISA. Whole-virus preparations were obtained by centrifuging 180 ml of virus (about 5,120 HAU/ml) at 24,000 rpm (∼105,000 × *g*) for 2 h in a Beckman SW32 Ti swing bucket rotor and a Beckman Optima L-100 XP ultracentrifuge. The pelleted viruses were resuspended in 1.2 ml of PBS. The resuspended virus protein concentration was titrated with a Pierce bicinchoninic acid (BCA) protein assay kit (ThermoFisher, Rockford, IL) and diluted to 50 μg/ml in coating buffer (0.1 M sodium carbonate, pH 9.6). The plates were coated with diluted virus (100 μl/well) at 4°C overnight. Then, the plates were blocked with 10% skim milk in PBS at room temperature for 2 h and washed twice with PBS containing 0.05% Tween 20 (PBST). The plates were incubated with test samples diluted in 2% skim milk in PBS at room temperature for 2 h. After washing 3 times with PBST, the plates were incubated with a horseradish peroxidase (HRP)-conjugated anti-mouse IgA antibody (Bethyl Laboratories, Montgomery, TX) or anti-mouse IgG antibody (ThermoFisher, Rockford, IL) at room temperature for 1 h and then washed 3 times with PBST. Then, 100 μl TMB solution (ThermoFisher, Vienna, Austria) was added to each well and incubated for 15 min at room temperature. The reaction was stopped by adding 3% H_2_SO_4_, and OD_450_ values were measured.

### Statistical analyses.

All data analyses were performed using GraphPad Prism software version 7 (GraphPad Software Inc., San Diego, CA). For multiple comparisons, two-way analysis of variance (ANOVA) was performed. Differences in survival curves were analyzed using the log-rank test. A *P* value below 0.05 was considered significant.
